# Benefits of Lactic Acid Bacteria Folate Synthesis in Foods and Risks Associated with Deficiency - a Review

**DOI:** 10.1007/s12602-026-11018-6

**Published:** 2026-04-24

**Authors:** Maria Fernanda Fernandes Siqueira, Khadija Bezerra Massaut, Patrícia Bohmer Radatz, Silvana de Souza Sigali, Igor Henrique de Lima Costa, Pedro Fernandes Viana, Graciela Volz Lopes, Wladimir Padilha da Silva, Ângela Maria Fiorentini

**Affiliations:** Department of Agroindustrial Science and Technology, Federal University of Pelotas, Pelotas, RS Brazil

**Keywords:** fermented foods, prevention, dysfunction, central nervous system, vitamin B9, bio-enriched

## Abstract

Folate (vitamin B9) is an essential micronutrient found in both animal and plant foods, but it is highly heat-sensitive, leading to loss of this nutrient during thermal processing. To overcome folate deficiency, many regulatory agencies recommend fortifying foods with folic acid (a synthetic form of the vitamin), although its long-term safety remains debated. A promising alternative approach involves bio-enriched fermented foods produced by lactic acid bacteria (LAB). These LAB-fermented products, such as dairy products (yogurt, cheese), cereals, and vegetables, have demonstrated potential to regulate intestinal homeostasis, alleviate inflammatory diseases, and mitigate neurodegenerative symptoms. This study is a systematic review that aims to assess the ability of LAB to synthesize folate, highlighting studied genera and their applications, benefits, and risks of deficiency. The species *Lactiplantibacillus plantarum* stands out in folate production, in vitro and in situ. The field of Food Science and Technology concentrates most of the research on folate, followed by the field of Microbiology. Furthermore, 93% of research, considering all categories, is correlated with Sustainable Development Goal (SDG). Three, which focuses on “Good Health and Well-Being”, and among the countries of origin, the United States leads in published research.

## Introduction

Folate is an essential micronutrient involved in numerous vital biological reactions and naturally appears as vitamin B9. Mammals lack the ability to synthesize vitamins, relying instead on exogenous sources such as diet and intestinal microbiota [1]. Folate consumed through food is converted into 5-methyltetrahydrofolate (5-MTHF), the active form of vitamin B9. This conversion allows the nutrient to be absorbed and utilized by the human body [2].

Folate is found in many foods, such as beans, dark green vegetables, citrus fruits, eggs, liver, meat, milk, seafood, oats, and nuts [3, 4]. Another strategy involves using lactic acid bacteria (LAB) to synthesize folate during the fermentation of a given food matrix. Many matrices (such as milk, cereals, and fruits), as well as containing a certain percentage of folate, enable fermentation by bacteria and increase the levels of natural folate in the fermented product [2, 5], constituting an important strategy to improve the nutritional composition of fermented products [6].

LAB are considered a group of microorganisms that share some common characteristics. They are Gram-positive staining, non-spore-forming, facultative anaerobic, catalase-negative and tolerant to acidic pH, and they produce lactic acid as a final product of carbohydrate metabolism [7]. Furthermore, they are Generally Recognized as Safe (GRAS), and many species have probiotic properties; therefore, they are widely used by the food industry [7, 8]. Additionally, they produce metabolites such as organic acids, bacteriocins, and vitamins. Among these vitamins, vitamin B9 stands out as an important nutrient for women of childbearing age, known for its role in preventing neural tube defects in fetuses and, in the general population, for reducing homocysteine levels (a metabolic indicator of folate deficiency). High levels of homocysteine can cause neuropsychiatric disorders [9]. Folate serves as an important cofactor for metabolic enzymes involved in biochemical pathways such as DNA replication and repair, as well as the biosynthesis of molecules like nucleotides, amino acids, and other vitamins [10–12]. Furthermore, besides their presence in food, LAB that survive gastrointestinal barriers can colonize the intestinal region and synthesize folates, which are then absorbed and utilized by the host through specific mechanisms [1].

Several genera of LAB such as *Lactobacillus*,* Streptococcus*,* Lactiplantibacillus*,* Limosilactobacillus*,* Lacticaseibacillus* and *Pediococcus*, are reported as having the ability to synthesize folate, both intracellularly and extracellularly [12–14]. The ability to produce folate is extremely strain-dependent and occurs via the *de novo* pathway from the availability of guanosine triphosphate (GTP), para-aminobenzoic acid (PABA), and glutamate. The combination of the pteridine fraction of GTP with PABA results in the tetrahydrofolate-polyglutamate (THF-polyglutamate) folate form, which is the main structure of all other existing folate forms [15–17].

Due to the high incidence of individuals with folate deficiency, many countries have adopted mandatory food fortification programs: currently, about 70 countries have implemented mandatory fortification of flour with folic acid [18]. However, it is important to consider that high folic acid intake can lead to adverse side effects, such as masking the symptoms of vitamin B12 deficiency and potentially promoting colorectal cancer or deactivating certain liver enzymes [13, 19]. These effects are not observed with natural folates; therefore, foods enriched with vitamin B9 from microbial production may be a promising alternative to folic acid supplementation [10].

The World Health Organization (WHO) and the Food and Agriculture Organization of the United Nations (FAO) recommend that most of the population, including adolescents, adults, and elderly individuals, should consume 400 µg of folate per day as part of their recommended nutrient intakes (RNIs). Infants and young children, on the other hand, require 65–300 µg of folate daily to meet their folate needs [20].

The use of vitamin-producing microorganisms, especially LAB, presents a natural and economically viable alternative to fortification with chemically synthesized pseudo-vitamins [21–23]. Although milk and dairy products contain folate (20 to 50 µg/L), these concentrations are not considered significant due to pasteurization and Ultra High Temperature (UHT) processing, which can reduce vitamin levels. A food is considered a good source of folate when it contributes at least 15% of the recommended daily intake [24].

This study aims to systematically review the capacity of LAB used in folate (vitamin B9) biosynthesis in fermented foods, exploring the main genera and strains studied, as well as their technological and functional applications. The review aims to contribute to the advancement of knowledge in the field of Food Science and Technology, with a focus on natural, safe, and sustainable solutions for the nutritional enrichment of fermented foods.

## Methodology

### Study Design

The search for articles was conducted in the Clarivate Analytics ISI – Web of Science (WoS) database, which was chosen due to its extensive collection of studies, with 254 subject categories where articles are indexed according to their content. This database has been used as a research and data collection tool in various academic fields [25]. With a rigorous selection process for indexing high-quality articles. Furthermore, WoS has been explored in studies focused on Food Science and Technology, allowing research gaps to be identified [26, 27].

To conduct the searches, some preliminary tests were performed in WoS to define a search strategy that would allow us to mine articles aligned with the study’s hypothesis. Therefore, the following inputs were defined for pattern recognition in articles: (folate* or folic acid*) and (lactic acid bacteria* or LAB).

### Inclusion and Eligibility Criteria

Pattern recognition yielded 346 results, which were screened based on inclusion criteria such as only primary source (experimental) articles, a 10-year time frame (2013 to 2023), and articles written in English. After this step, the selected articles were checked for relevance to the topic by reviewing the title, keywords, and abstract, in that order. Consequently, 194 articles were selected as eligible for bibliometric analysis.

### Bibliometric Analysis

#### Bibliometric Indicators

The experimental articles were previously evaluated using the analysis tools available in WoS. Based on this prior assessment, article metadata were imported to develop bibliometric indicators of publication dynamics, indexing of articles in the 10 main WoS categories, geographic location of scientific publications according to the 10 leading countries, and association of publications with the United Nations Sustainable Development Goals (SDG) [28]. The number of citations per year and H-Index metrics were also evaluated.

#### Conceptual Structure Analysis

Metadata from articles imported from WoS were analyzed using VOSviewer (version 1.6.19) and RStudio (version 2023.06.1) software to evaluate the conceptual structure of the metadata set. A co-word analysis was developed based on the articles’ Keywords Plus, using a walk trap clustering algorithm.

In VOSviewer, Keywords Plus were normalized using the association method, and the total count method was used to generate a bibliometric co-occurrence map in a low-dimensional Euclidean space with a minimum of 50 nodes. In RStudio, the *bibliometrix* package was used to conduct the analyses [29]. The accumulated occurrence of Keywords Plus over time was evaluated. Furthermore, Multiple Correspondence Analysis (MCA) was used to investigate the level of information alignment between articles and evaluate the formation of clusters, according to the following pre-definitions: analysis of 30 terms and formation of up to 4 clusters. In the thematic evolution analysis, an inclusion index weighted by the occurrence of words and with two cutoff points in 2018 and 2021 was considered, allowing us to evaluate the terms of interactions during the periods 2013 to 2018, 2019 to 2021, and 2022 to 2024. This highlighted the multidisciplinary nature of the topic under study and enabled the evaluation of advances related to lactic acid bacteria and folate.

### Outline Structure

Considering that publications on folate are mostly associated with SDG 03 – “Good Health and Well-Being,” the 198 selected articles were divided into three topics, with the purpose of directing and elucidating the issues surrounding SDG 03. Topic 1: Lactic acid bacteria and their folate biosynthesis capacity; Topic 2: Folate: its metabolic function, absorption and ingestion vs. pathologies caused by a deficiency; Topic 3: Bio-enriched foods with folate produced by lactic acid bacteria. In addition to the 198 articles, reviews cited in the database formed were also used, with the purpose of discussing and also making some information clearer.

## Research Output Trends and Thematic Evolution

### Bibliometric ors and Conceptual StructureIndicat

**Fig. 1:**
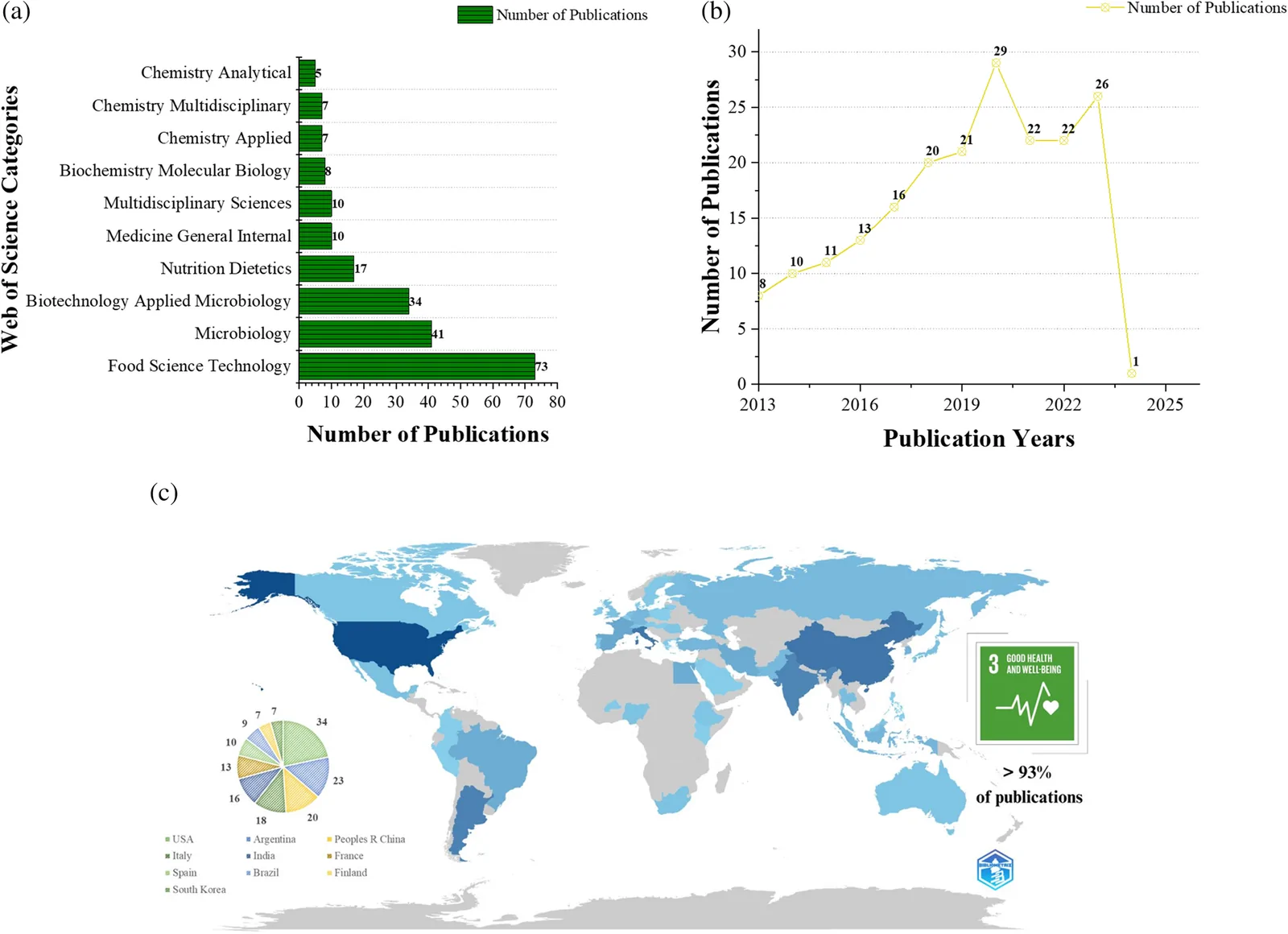
Bibliometric indicators of publications related to the main Web of Science categories (**a**), publication dynamics (**b**), geographic location, and UN SDG (**c**) associated with LAB and folate

Considering the 10 main WoS categories in which articles related to lactic acid bacteria and folate were indexed (Fig. 1a), Food Science Technology stands out with 73 publications, making it the category with the largest number of publications, followed by Microbiology (41 publications), Biotechnology Applied Microbiology (34 publications), Nutrition Dietetics (17 publications), Medicine General Internal (10 publications), Multidisciplinary Sciences (10 publications), Biochemistry Molecular Biology (eight publications), Applied Chemistry (seven publications), Chemistry Multidisciplinary (seven publications), and Chemistry Analytical (five publications).

The number of publications in the time frame used (Fig. 1b) for the present study (2013 to 2024) was variable, with a linear trend between 2013 and 2018 and no defined trend between 2019 and 2024. The year 2020 presented 29 publications, and 2023 obtained 680 citations, which is the year with the highest number of publications and citations, respectively. Furthermore, for the set of articles identified with pattern recognition, an H-Index of 30 was observed, indicating that at least 30 of the articles found were cited 30 times or more.

The indicator of number of publications by geographic distribution (top ten) and association with the UN SDGs (Fig. 1c) highlighted the USA with 34 publications, making it the country with the highest number of publications, followed by Argentina (23 publications), People’s Republic of China (20 publications), Italy (18 publications), India (16 publications), France (13 publications), Spain (10 publications), Brazil (nine publications), Finland (seven publications) and South Korea (seven publications). Furthermore, the publications were mainly associated (> 93%) with “SDG 03 - Good Health and Well-Being” and, in the minority, with “SDG 02 - Zero Hunger” (2.5%), “SDG 12 - Responsible Consumption And Production” (0.5%), “SDG 06 - Clean Water And Sanitation” (0.5%), “SDG 13 - Climate Action” (0.5%) and “SDG 15 - Life On Land” (0.5%).

**Fig. 2:**
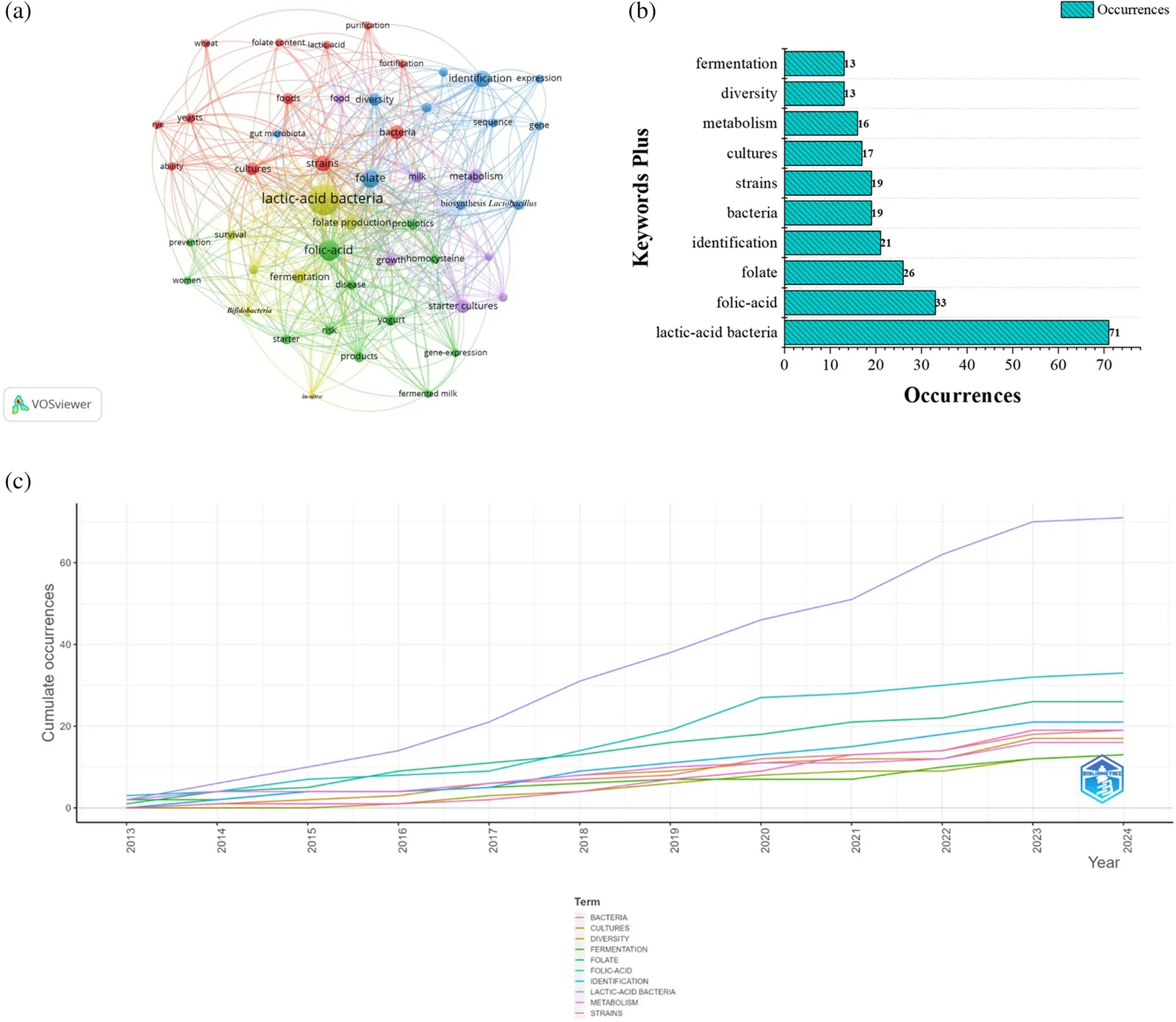
Bibliometric map of co-occurrence of Keywords Plus (**a**), its main terms (**b**), and accumulated occurrence (**c**) related to LAB and folate

The bibliometric co-occurrence map of Keywords Plus (Fig. 2a) was centered on “lactic acid bacteria” (71 occurrences; Fig. 2b), forming five clusters, with the red cluster being the most representative of the metadata set. Its conceptual structure addresses the investigation of different strains of LAB for folate production, focusing on purification strategies for this compound for technological application in the fortification of food products.

In the green cluster (Fig. 2a), terms that refer to products where folate is present or that can be fortified are highlighted, such as “yogurt” and “fermented milk”. The presence of this vitamin in foods for daily consumption or quick consumption is a strategy to reduce the risk of diseases, act in long-term prevention, and provide the well-being of consumers, corroborating SDG 03 [30]. This highlights the importance of food fortification at a global level, contributing to the UN 2030 agenda on infant mortality and health equality.

The blue cluster addresses the identification of folate-producing LAB with a molecular approach in gene sequencing. This approach is essential in the identification of species of microorganisms, allowing the distinction and appropriate choice of LAB in fermentative and folate production processes. The purple cluster highlights the use of starter cultures in fermentative processes in milk to obtain derived products, including the identification of metabolites involved in the process.

The accumulated occurrence for the main terms in the time frame from 2013 to 2024 was investigated (Fig. 2b). It is observed that for “lactic acid bacteria,” there was an increasing linear behavior between 2013 and 2024, with the number of accumulated occurrences > 70. All other terms showed increasing behavior, maintaining their accumulated occurrence ≤ 20, except for the term “folate,” which showed accumulated occurrence ≥ 30. These findings suggest that studies with LAB have been increasing during this period, with a focal point on folate production.

**Fig. 3:**
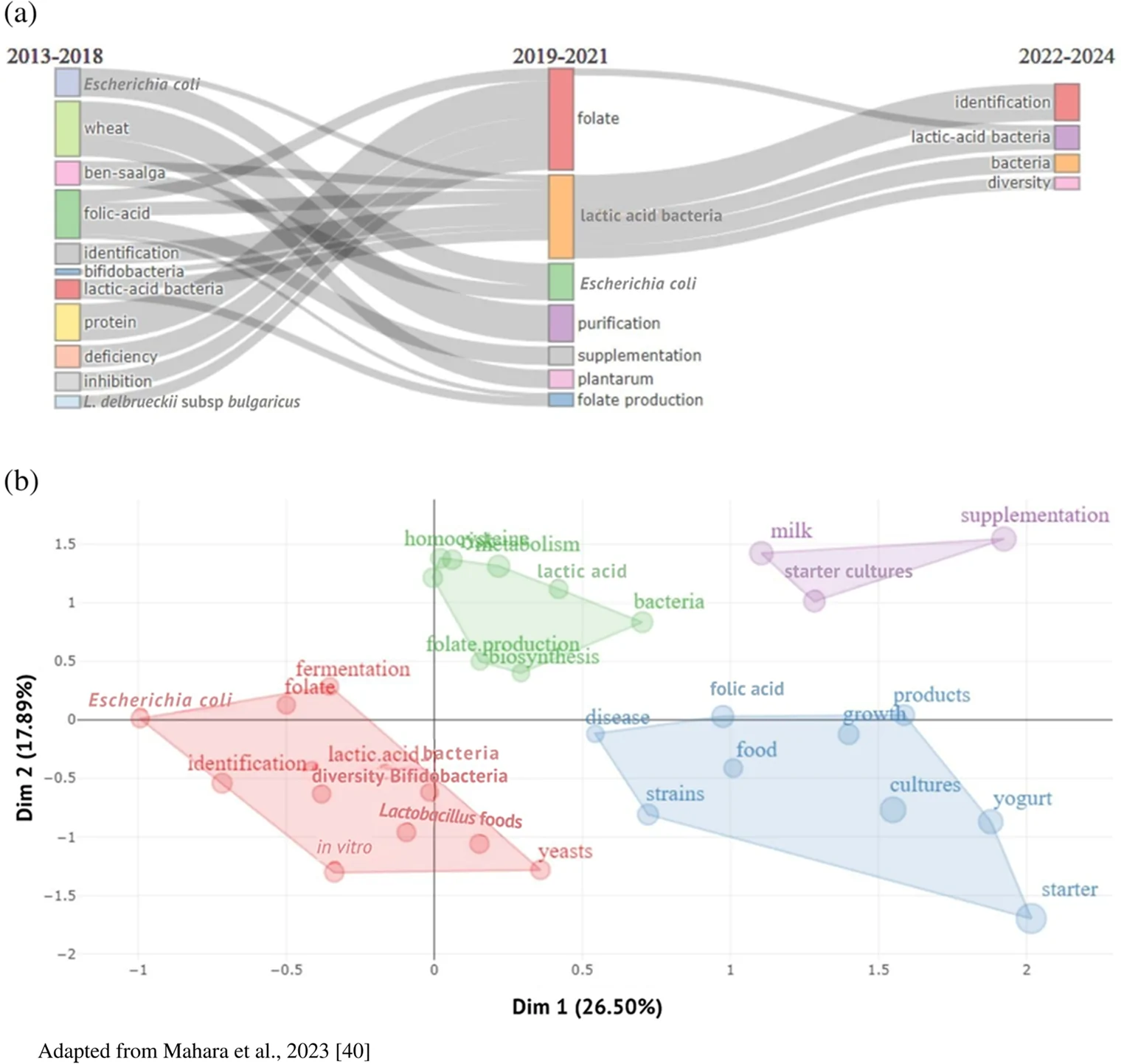
Thematic evolution in three time periods (**a**) and Analysis of Multiple Match (**b**) for Keywords Plus related to LAB and folate

In the thematic evolution (Fig. 3a), it is observed that between 2013 and 2018, the use of LAB was concentrated in different research segments, including antagonism to pathogenic bacteria in vitro and in situ, use of standardized strains in the investigation and optimization of fermentative processes of food products, and bioprospecting studies to identify bacteria with potential for use in the food industry. During this period, there were investigations involving folic acid; however, the number of studies was limited. This scenario changed between 2019 and 2021, with an increase in research on folate, such as studies on vitamin deficiency in individuals and on folate production. This period was marked by the pandemic caused by the SARS-CoV-2 virus, with periods of restrictions on movement and difficulties in accessing food possibly contributing to folate deficiency among consumers [31]. Between 2022 and 2024, there was a noticeable trend in bioprospecting LAB of interest in food for folate production. The increase in studies focused on the production of this molecule aligns with the trends observed in cumulative occurrence (Fig. 2c).

The ACM (Fig. 3b) revealed the formation of four clusters in the factorial plan, with an information alignment level upper limit of 44% across dimensions (Dim 1 and Dim 2) in the identified articles using pattern recognition. The coordinates of the red cluster positioned it in a negative region of the factorial plane in both dimensions, suggesting that studies involving antagonism against pathogenic bacteria such as *Escherichia coli*, LAB prospection for fermentative processes, and folate production had a low occurrence in Keywords Plus. The other clusters (green, blue, and purple) show a higher number of studies involving LAB in the production of dairy products, participation in biosynthesis processes, folate production, and supplementation of products with folic acid.

### Lactic Acid Bacteria and their Folate Biosynthesis Capacity

The use of LAB for folate production is a promising strategy that can help us achieve the SDGs outlined in the UN 2030 agenda. One of the objectives of SDG-03 is to “Reduce by one third premature mortality from non-communicable diseases through prevention and treatment and promote mental health and well-being.” A balanced and healthy diet is known to contribute significantly to preventing chronic diseases and brain disorders [32]. In this context, LAB can contribute due to their ability to produce various metabolites of interest, such as organic acids, bioactive peptides, fatty acids, and vitamins (complex B, A, and K) [33].

In recent years, several studies have explored the technological and probiotic properties that LAB may offer, including enzymatic activities (proteolytic, lipolytic), treatment of obesity-related cardiovascular diseases, maintenance of intestinal microbiota, and immunomodulatory effects [34–38]. Among the B-complex vitamins, folate (vitamin B9) is considered an essential micronutrient and acts as a cofactor in several metabolic reactions in the body [39]. The main genera of LAB capable of synthesizing this vitamin are *Streptococcus*, *Lactococcus*, and *Lactobacillus*.

Folate is a conjugated compound formed by a pteridine ring and para-aminobenzoic acid (PABA). It is present in various forms, characterized both by the level of oxidation of the pteridine ring and according to the substituents attached to N5 and/or N10 positions [40]. If pteridine is completely oxidized, folate is called folic acid (FA); if it is partially reduced, it is called dihydrofolate (DHF); if it is completely reduced, it is called tetrahydrofolate (THF). THF can have derivatives that act as cofactors in receiving and donating carbons in methylation reactions, as in the synthesis of DNA and amino acids [41]. This derivation occurs through the coupling of substituents to N5 and/or N10 positions, generating different folate derivatives, such as 5-methyltetrahydrofolate, 5,10-menthenyltetrahydrofolate and 5-formyltetrahydrofolate, 5,10-methylenetetrahydrofolate.

Table 1 presents the LAB isolates capable of synthesizing folate in vitro based on available studies according to the search criteria used. It should be noted that the scientific nomenclature of some LAB species has been recently updated [42]. However, for consistency with the publications reviewed, the names in Table 1 have been maintained as per the sources. The reviewed articles date back to 2017, and analysis of Fig. 1a reveals that from that year onwards, research on the folate production capacity of LAB began to be developed and published more prominently.

**Table 1:** List of lactic acid bacteria (LAB) evaluated for folate synthesis capacity in vitro

Lactic acid bacteria	Main result of folate production	Quantification method	Reference
*Lactobacillus bulgaricus*, *Lactococcus lactis*, *Pediococcus pentosaceus*,* Lactobacillus rhamnosus*, *Lactobacillus casei*	*Lactobacillus plantarum* − 3.72 µg/mL	HPLC	Zhang et al.(2020) [43]
*Lactobacillus pentosus*	*Lactobacillus pentosus* ZFM94–17.2 µg/L	MA	Ye et al. *(*2020) [93]
*Lactobacillus plantarum*,* Lactobacillus casei*, *Lactobacillus paracasei*,* Lactobacillus gasseri*	*Lactobacillus plantarum* GM-12–170 ng/dL	SPT	Tulumoğlu et al. (2022) [94]
*Streptococcus thermophilus*	*Streptococcus thermophilus* M17PTZA496–174.67 ng/mL	MA	Tarrah et al. (2018) [95]
*Lactobacillus plantarum*	*Lactobacillus plantarum* P2R3FA − 43 µg/L	MA	Tamene et al. (2022) [85]
*Enterococcus mundtii*, *Lactobacillus pentosus*, *Pediococcus acidilactici*, *Enterococcus faecalis*, *Enterococcus gallinarum*, *Enterococcus durans*, *Pediococcus pentosaceus*,* Lactobacillus pentosus* *Lactobacillus fermentum*, *Lactobacillus plantarum*, *Lactobacillus brevis*	*Enterococcus mundtii* ES63–34 ng/mL *Lactobacillus pentosus* ES124–33 ng/mL	MA	Salvucci et al. (2016) [96]
*Lactococcus lactis* subsp. *lactis*	*Lactococcus lactis* subsp. *lactis* C195–595 ng/mL *Lactococcus lactis* subsp. *lactis* C173–58.0 ng/mL	MA	Sabo et al. (2020) [97]
*Lactobacillus plantarum*	*Lactobacillus plantarum* CECT 8965 − 32.43 ng/mL *Lactobacillus plantarum* CECT8962–54.66 ng/mL	MA	Rodrigo-Torres et al. (2019) [98]
*Lactobacillus plantarum*	*Lactobacillus plantarum* JA51–9.03 µg/mL	HPLC	Park et al. (2014) [99]
*Lactobacillus rhamnosus*, *Lactobacillus brevis*	*Lactobacillus rhamnosus* IFM4–35 ng/mL	MA/ HPLC	Panda et al. *(*2018) [86]
*Lacticaseibacillus rhamnosus*, *Lactiplantibacillus plantarum*,* Limosilactobacillus fermentum*, *Leuconostoc mesenteroides*, *Pediococcus acidilactici*, *Lactobacillus kefiri*	*Lacticaseibacillus rhamnosus* R23–98.37 µg/mL *Limosilactobacillus fermentum* JK13–85.67 µg/mL	HPLC	Mahara et al. (2023) [100]
*Lactobacillus brevis*, *Lactobacillus casei*, *Lactobacillus fermentum*, *Lactobacillus sakei*, *Lactobacillus farciminis*, *Leuconostoc mesenteroides*	*Lactobacillus sakei* − 70 ng/mL	MA	Jiménez et al. (2018) [46]
*Lactobacillus fermentum*, *Lactobacillus plantarum*, *Lactobacillus rhamnosus*	*Lactobacillus fermentum* WTS4–0.9 µg/mL	HPLC	Hati et al. (2019) [101]
*Lactobacillus fermentum*, *Lactobacillus plantarum*	*Lactobacillus plantarum* − 97 ng/mL	MA	Greppi et al. (2017) [16]
*Lactococcus hircilactis*, *Lactococcus laudensis*	*Lactococcus hircilactis* − 15.96 µg/mL	MA	Tidona et al. (2018) [75]
*Lactobacillus sakei*, *Lactobacillus fermentum*, *Lactobacillus* sp. *Lactobacillus casei*, *Lactobacillus brevis*	*Lactobacillus sakei* CRL2210–1484 ng/g *Lactobacillus sakei* CRL2209–730 ng/g	MA	Mosso et al. (2018) [12]
*Lactobacillus plantarum*	*Lactobacillus plantarum* CKR26–74.20 µg/L	HPLC	Bhagya et al. (2018) [102]

*MA* Microbiological Assay, *SPT *Spectrophotometry, *HPLC* High Performance Liquid Chromatograph

It is noteworthy that *Lactobacillus* predominates among the most cited genera capable of synthesizing folate, with *Lactobacillus plantarum* (now *Lactiplantibacillus plantarum*) being particularly prominent. *Lactobacillus pentosus* was highlighted twice as the primary result for folate synthesis, along with *Lactobacillus rhamnosus* (now *Lacticaseibacillus rhamnosus*), *Lactobacillus sakei*, *Lactobacillus fermentum* (now *Limosilactobacillus fermentum*), *Lactococcus lactis* subsp. *lactis*, *Enterococcus mundtii*, *Streptococcus thermophilus*, and *Lactococcus hircilactis*, each mentioned once.

**Fig. 4:**
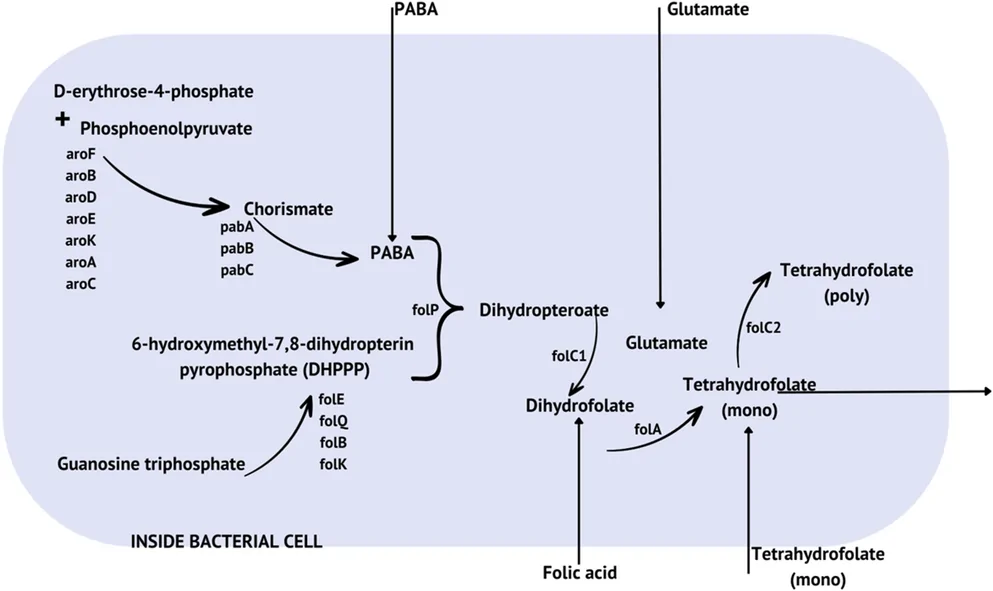
Biosynthesis folate by *de novo* pathway

LAB can synthesize folate by *de novo* pathway, that is, through the production of enzymes that modify simple molecules through changes in functional groups [1, 40]. The genes involved in *de novo* biosynthesis are: (*i*) related to the synthesis of chorismic acid (*aroB*,* aroD*,* aroE*,* aroK*,* aroF*,* aroA*,* aroC*); (*ii*) related to the synthesis of pABA (p-aminobenzoic acid) (*pabB*,* pabA*); (*iii*) related to the synthesis of DHPPP (6-hydroxymethyl-7,8-dihydropterin pyrophosphate) (*folE*,* folQ*,* folB*,* folK*) and (*iv*) related to the synthesis of THF-polyglutamate (tetrahydrofolate- polyglutamate) (*folP*,* folC folA)* [43]. Figure 4 illustrates the mechanism of *de novo* pathway folate production.

In a study where 12 LAB isolates were evaluated for the presence of DHPPP and pABA precursor genes related to folate synthesis (*aroB*,* aroD*,* aroE*,* aroK*,* aroF*,* aroA*,* aroC*,* pabB*,* pabA*,* folE*,* folQ*,* folB*,* folK*,* folP*,* folC*, and *folA*), it was observed that 80% of *L. plantarum* isolates had all 16 genes. The preservation of these 16 genes within the species would be a plausible justification for the occurrence of *L. plantarum* in reports in the literature in relation to folate synthesis [43]. However, not all LAB can synthesize the PABA precursor, with the same results being achieved by exogenous means, especially from the genus *Lactobacillus* [40]. This result is an indication that further studies regarding the *de novo* folate synthesis property are necessary.

The literature indicates that, unlike other LAB, the folate biosynthetic pathway in *Lactiplantibacillus plantarum* exhibits low sensitivity to the negative regulation exerted by exogenous folate or its precursors, so that the presence of these compounds in the medium does not significantly suppress endogenous vitamin production [17] This results in low stress resistance, which consequently leads to overproduction of the metabolite in question, as observed by Zhang et al. 2020, where the *L. plantarum* GSLP-7 V isolate showed increased folate production when subjected to stress caused by methotrexate, an analog of DHF [43]. Methotrexate resistance in *L. plantarum* strains was also reported by other authors more than 15 years ago, indicating that this topic still needs further study.

LAB have regulatory mechanisms to control folate biosynthesis efficiently; some studies highlight the feedback mechanism, where there is signaling through the final products, as in the case of the first step of folate biosynthesis, where the activity of the enzyme dihydropteroate synthase (DHPS) is involved in the conversion of DHPPP and PABA into Dihydropteroate (DHP) [44]. In a study carried out by Laiño et al. (2019), feedback inhibition of the *folK* gene was observed, which is overexpressed and then declines over a long period of fermentation, indicating that when folate production is sufficient, the activity of enzymes involved in folate biosynthesis can be repressed [45].

Obtaining THF (poly)-tetra-hydrofolate-polyglutamate occurs when the *folC*_*2*_ gene is undergoing repletion, and with this, glutamates present in the intracellular environment are added to the THF (mono)-tetrahydrofolate-monoglutamate fractions. This takes place through the action of the enzyme FPGS (expressed by the *folC*_*2*_ gene) responsible for lengthening the polyglutamate chain and consequently increasing the amount of extracellular folate [40], considering that the longer the polyglutamate chain, the more easily is THF retained inside the cell [46]. In a study carried out by Serrano-Amatriain et al. (2016), overexpression of the *folC*_*1*_ and *folE* genes was able to increase the production of extracellular folate by up to eleven-fold [47], which is an indication that folate-producing bacteria necessarily must possess the *folC* gene, which may also be present in the form of two separate genes, *folC*_*1*_ and *folC*_*2*_, since both are involved in the initiation of the transformation of folate precursors into THF [48, 49]. Table 2 contains a compilation of the genes involved in the description of *de novo* synthesis and precursors in the respective steps of the metabolic pathway.

**Table 2:** Steps of *de novo* folate biosynthesis, respective genes involved and corresponding precursors

Initial molecule	Genes involved	Enzyme	Essential precursor
	First stage of biosynthesis		
Phophenol pyruvate D-erythrose-phosphate	*aroF*	3-deoxy-d-arabino-heptulosonate-7-phosphate synthase, DAHPS, EC 2.5.1.54	Chorismic acid
	*aroB*	3-dehydroquinate synthetase, DHQS, EC 4.2.3.4	
	*aroD*	3-dehydroquinate dehydratase, DHQD, EC 4.2.1.10	
	*aroE*	shikimate 5-dehy-drogenase, SDH, EC 1.1.1.25	
	*aroK*	shikimate kinase, SK, EC 2.7.1.71	
	*aroA*	5-enol-pyruvylshikimate-3-phosphate synthase, EPSPS, EC 2.5.1.19	
	*aroC*	chorismate synthase, CS, EC 4.2.3.5	
Chorismic acid	*pabA*	aminodeoxychorismate synthase component I, EC 2.6.1.85	PABA
	*pabB*	aminodeoxychorismate synthase component II, EC 2.6.1.85	
	*pabC*	4-amino-4-deoxychorismate lyase, EC 4.1.3.38	
	Second stage of biosynthesis		
Guanosine triphosphate (GTP)	*folE*	GTP cyclohydrolase I, EC 3.5.4.16	7,8-Dihdroneonpterintriphosphae
7,8-Dihydroneopterintriphosphae	*folQ*	dihydroneopterin triphosphate pyrophosphohydrolase, DHNTP, EC 3.6.1	7,8-Dihydropterinmonopohsosphate
7,8-Dihydropterinmonopohsosphate	Unidentified gene	-	7,8-Dihdroneopterin
7,8-Dihdroneopterin	*folB*	dihydroneopterin aldolase, EC 4.1.2.25	6-hydroxmethyl-7,8-fihdropterin
6-hydroxmethyl-7,8-fihdropterin	*folK*	hy-droxymethyl dihydropterin pyrophosphokinase (HPPK), EC 2.7.6.3	DHPPP
	Third stage of biosynthesis		
PABA DHPPP	*folP*	dihydropteroate synthase, DHPS, EC 2.5.1.15 dihydrofolate synthase, DHFS, EC 6.3.2.12	Dihydropteroate
Dihydropteroate	*folC*_*1*_	dihydrofolate synthase, DHFS, EC 6.3.2.12	Dihydrofolate
Dihydrofolate	*folA*	dihydrofolate reductase, DHFR, EC 1.5.1.3	THF-monoglutamate
THF-monoglutamate	*folC*_*2*_	folylpolyglutamate synthase, FPGS, EC 6.3.2.17	THF-polyglutamate

Adaptation from Mahara et al. (2023) [40]

*PABA* Para-aminobenzoic acid, *DHPPP* 6-hydroxymethyl-7,8-dihydropterin pyrophosphate, *THF *Tetrahydrofolate

### Folate: its Metabolic Function, Absorption and Ingestion vs. Pathologies Caused by a Deficiency

Adequate daily folate intake is of paramount importance for health, as insufficient intake or irregular consumption can trigger numerous disorders, especially those related to the central nervous system. Folate is responsible for maintaining homeostasis in the central nervous system and is also involved in the regulation of essential cellular processes [50, 51]. Therefore, alterations in its concentration and transport in the brain can induce various neurological disorders, particularly in children [52–54]. Folate absorption is known to depend on specific transport mechanisms to permeate epithelial cells; the liver (in humans) and the intestinal mucosa (in rodents) represent the main pathways for folate metabolism and absorption, although dietary folate can also be reabsorbed by the renal system [55, 56].

In mammals, the three folate transport pathways characterized are Folate Receptors (FR), Reduced Folate Carrier (RFC), and the Proton-Coupled Folate Transporter (PCFT). FRs and PCFT are primarily involved in transporting folate to the brain at the choroid plexus, while RFC is responsible for folate transport into cells of peripheral tissues, including the brain parenchyma at the blood-brain barrier [47, 53]. Abnormalities related to these pathways directly involve folate deficiency and associated pathologies. The main pathologies include neural tube defects, hereditary folate malabsorption, cerebral folate deficiency, Kearns-Sayre syndrome, autistic spectrum disorders, cerebral folate deficiency during early childhood, development of fetal anomalies, neuropathies, neurodevelopmental disorders, macrocytic anemia and elevated levels of homocysteine in plasma and brain tissues, which can trigger cardiovascular and inflammatory diseases and schizophrenia [51, 52, 57, 58].

However, it is important to highlight that in some cases, folate deficiencies can occur for other reasons, such as low intake (typical in older people, children, alcoholics, and socially vulnerable people), malabsorption problems (associated with inflammatory bowel diseases such as Crohn’s and ulcerative colitis, people with celiac disease and those undergoing bariatric surgery), physiological changes that increase the need for folate (such as pregnancy, lactation and pathologies such as anemia, cancer, rheumatoid arthritis and psoriasis) or use of chemical substances (such as anticonvulsants, drugs and alcohol) [50, 59].

Folate naturally present in foods is found in the forms THF, 5-methyl THF, and 10-formyl THF, which are more easily absorbed by mammalian cells, mainly in the colon. Furthermore, several bacterial strains of the intestinal microbiota (such *Lactobacillus reuteri* and *Bifidobacterium longum*) can synthesize and secrete THF, which can be absorbed in the intestinal epithelium. The folate production process, which involves interactions between host and microorganism, occurs when polyglutamylated tetrahydrofolates are synthesized by the human intestinal microbiota. Enzymes such as human gamma-glutamyl hydrolase (GGH) and gamma-glutamyl transferase (GGT) remove portions of glutamate, converting folates into monoglutamylated forms, which other bacteria can then absorb in the intestinal mucosa. Human enterocytes can absorb folate by activating folate receptors (Frα or Frβ) or via the folate transporters (RFC or PCFT) [60].

**Fig. 5:**
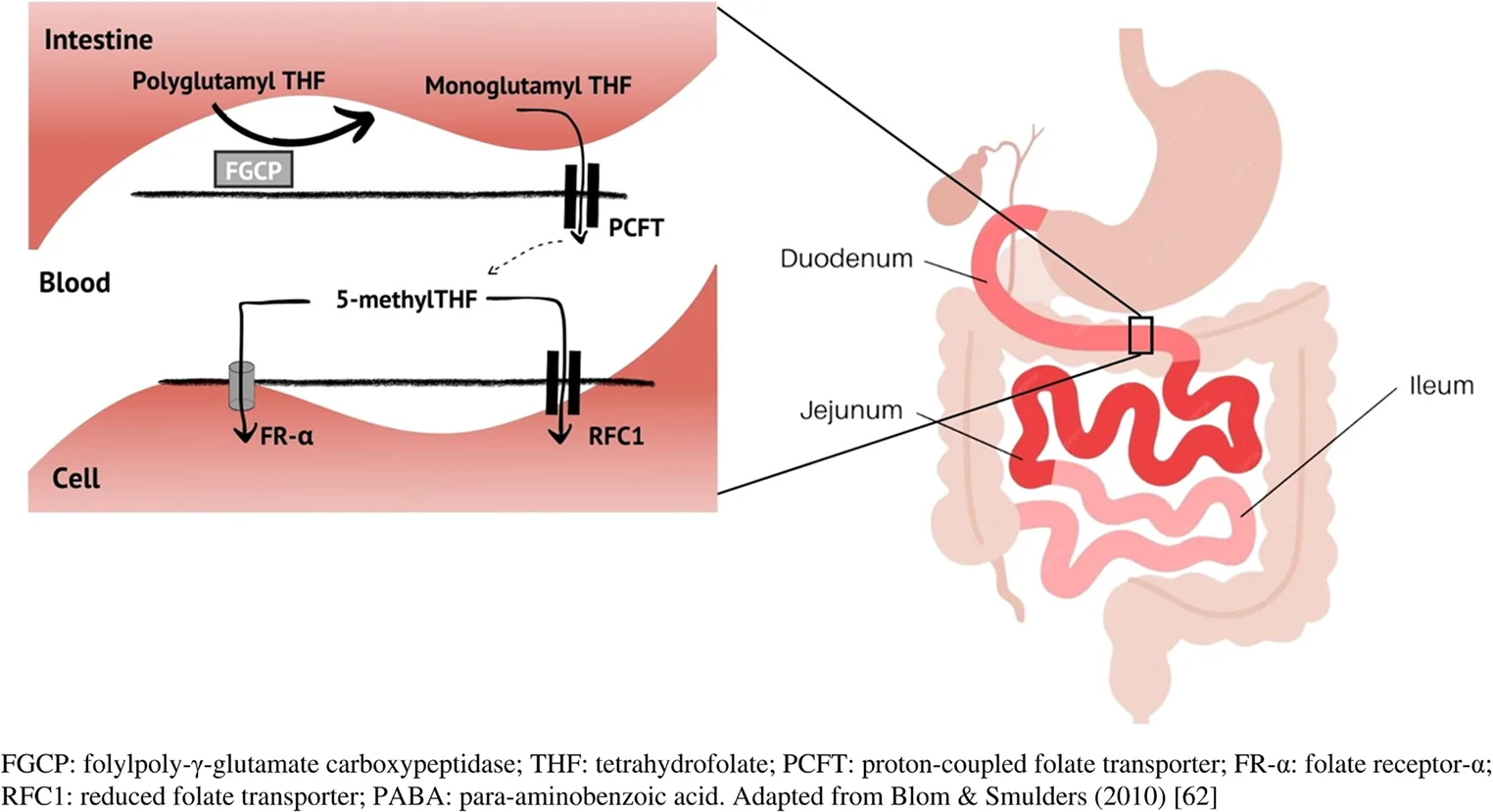
Region of absorption and transport of folate through the blood to the cells in the small intestine

The main site of folate absorption in the small intestine is in the duodenum and upper part of the jejunum (Fig. 5), where the polyglutamate form is hydrolyzed to the monoglutamate form by the action of the enzyme folylpoly-γ-glutamate carboxypeptidase (FGCP), located on the apical membrane of enterocytes in the intestinal mucosa and which is encoded by the glutamate carboxypeptidase II (GCPII) gene. Monoglutamates are subsequently absorbed in the small intestine by the high-affinity proton-coupled folate transporter (PCFT). In plasma, the main form in which folate is found is 5-methyl THF, which can be transported to the cell by folate receptor-α (FR-α) or reduced folate transporter (RFC) [58, 61, 62].

Folate absorption in the intestine is influenced by the presence of folate produced or metabolized by microorganisms in the small intestine; additionally, folate produced by bacteria can be captured along the entire length of the intestine [61]. Studies indicate that folate supplementation can restore serum levels of the vitamin and also promote improvement in deficits associated with its deficiency, whether through food intake, vitamin supplementation, or supplementation with folate-producing microorganisms.

A study with BALB/c mice evaluated the bioavailability and effects on the intestinal mucosa after ingestion of milk fermented with folate-producing *S. thermophilus* 34v and *L. plantarum* 16cv. Furthermore, the animals that received the fermented milk showed an increase in hemoglobin, hematocrit, and red blood cell levels. Histological analysis of the small intestine revealed a better relationship between villus height and crypt depth compared to folate-depleted animals. According to the authors, folate deficiency can promote changes in the intestinal mucosa. Because this vitamin is involved in the process of cell proliferation and differentiation, when folate administration was resumed, there was a beneficial effect on the mucosa, helping to maintain its integrity and, consequently, improving the animals’ intestinal health [9].

Laiño and co-workers produced fermented milk bio-enriched with folate using *L. bulgaricus* CRL871, *S. thermophilus* CRL803, and *S. macedonicus* CRL415. The study aimed to evaluate folate and homocysteine levels in BALB/c mice in a folate depletion/repletion model. Data from the study indicate that animals that received bio-enriched fermented milk had levels of folate in plasma and whole blood five times higher compared to depleted mice. Furthermore, folate replacement in groups where there was total restriction of the vitamins demonstrated a significant reduction in plasma homocysteine levels, reaching values like those in the control group, and these data were also associated with an increase in the time of folate replacement in the groups [63]. Therefore, the consumption of milk fermented with folate-producing starter cultures is not only effective in improving the status of the vitamin but can also prevent its deficiencies. As these starter cultures are easily absorbed, it can be said that folates produced from microbial fermentation are more effective than supplemented folic acid.

Similarly, from an in vivo model of folate deficiency, Zhang et al. (2020) sought to elucidate the effects of the folate-producing strain and bio-enriched yogurt. They showed that the administration of both LAB and yogurt had a synergistic effect on the recovery of serum folate levels and modulation of the animals’ intestinal microbiota, increasing its diversity; besides, they were also able to reduce homocysteine levels significantly in the supplemented animals [43].

Plasma homocysteine concentration and fecal microbiota composition of C57BL/6J mice were altered after supplementation with different dairy matrices (milk, fermented milk, and fermented milk bioenriched with folate by fermentation with *S. thermophilus* 563, *L. delbrueckii* subsp. *lactis* 1021, and *L. helveticus* 989) in a study conducted by Zinno et al. (2019). According to the results obtained, all matrices tested were effective in reducing plasma homocysteine levels in the treated animals, also resulting in greater diversity of the fecal microbiota. However, bioenriched fermented milk restored levels similar to those of the control group, both in homocysteine and S-adenosyl-methionine levels, and reduced the dysfunction triggered by folate deficiency, which was restored only when the ingested folate levels were higher than the concentration in fermented milk and dairy diets [64].

Furthermore, Tamene et al. (2019) evaluated the ability to restore normal blood folate status after depletion in an in vivo model. It was found that although the total folate consumption of animals that received the *L. plantarum* P2R3FA strain (175 ± 21.5 µg) was lower than the total folic acid content consumed by the control group (1398 ± 129.2 µg), the microorganism was able to increase folate levels in animals. This demonstrated that bacterial folate was bioavailable in the gastrointestinal tract (GIT), considering that serum folate levels after 30 days of administration rose to 18.4 nmol/L, while in the animals in the control group the value was 80 nmol/L, and in the depletion group the levels were above 10 nmol/L [65].

The anti-inflammatory properties of a combination of vitamin-producing LAB (folate and riboflavin) and immunomodulators, composed of *L. plantarum* CRL2130 and *S. thermophilus* CRL808 and *S. thermophilus* CRL807, were evaluated in BALB/c mice, during the remission period of an experimental model with provoked colitis. Likewise, the potential for reducing side effects caused by anti-inflammatory medication during the administration of the strains was evaluated. The results obtained demonstrate that animals treated with the LAB combination had greater weight gain at the end of the experiment compared to those treated with the medication alone. In the same way, the data obtained in the histological analysis of the intestine show that the animals that received a combination of LAB, with or without the drug, contained unaltered epithelia, presenting the lowest scores of histological damage, and the levels of cytokines (IL6 and TNF-α) were also significantly lower in the groups that received microorganisms. Therefore, the authors concluded that the combination of strains used, in addition to the beneficial effect on animals in remission, did not influence the primary treatment, with anti-inflammatories, in an in vivo model of colitis, improving the animals’ quality of life, making it a potential adjuvant in the treatment of this pathology [59].

On the other hand, Perez et al. (2020), using the same combination of *L. plantarum* CRL2130, *S. thermophilus* CRL808 and *S. thermophilus* CRL807, evaluated the neuroprotective effect of the strains in an in vivo model of Parkinson’s disease. Regarding motor deficiencies, an improvement in the animals’ performance was observed after receiving the strains, with a status statistically similar to that observed in the control group. Furthermore, the animals supplemented with the LAB combination had significantly higher IL10 serum levels than the groups that did not receive LAB. The authors showed that the combination of LAB was able to promote an improvement in the animals’ motor behavior, which is typically impaired by Parkinson’s disease. According to the results, they associated the effects with different mechanisms of action, such as the production of B vitamins and their antioxidant properties and the modulation of the host immune response [66]. However, other mechanisms, such as the modulation of the intestinal microbiota, have not been elucidated, and additional studies are needed.

The folate-producing strain *L. sakei* LZ217 was evaluated for interaction effects on the intestinal microbiota. It was observed that in the in vitro fecal fermentation test, there was a positive interaction because the microorganism was capable of altering the diversity of the fecal microbiota, besides producing butyric acid, a short-chain fatty acid associated with the maintenance of intestinal homeostasis. However, it is important to highlight that the initial composition of the fecal microbiota is a factor that should be explored in future studies, considering that its diversity may be closely related to the interaction between microorganisms and the production of their metabolites [67].

Finally, from previous studies, it is observed that supplementation with folate from the ingestion of folate-producing LAB strains or fermented foods bio-enriched with the vitamin allows the restoration of serum folate levels, the modulation of the fecal microbiota, and the regulation of intestinal homeostasis, also improving inflammatory diseases. Therefore, the use of the exogenous form of folate may be a promising alternative in the treatment and prevention of diseases associated with folate deficiency and an alternative to folic acid supplementation in foods.

### Foods Bio-enriched with Folate Produced by Lactic Acid Bacteria

Investigations are reported on the ability of LAB to produce folate in different food matrices, but there is no indication of the origin or initial focus of studies on folate production by LAB.

Folate is currently produced primarily through chemical synthesis; however, a viable alternative to synthetic vitamins for fortifying foods is through bio-production by LAB. Besides the economic advantage, this practice also adds value to the product and increases competitiveness in the food industry [68]. Among the foods bio-enriched with folate, both animal and plant-based products have shown a promising future, as evidenced by studies conducted by various researchers using different species of LAB.

Bovine milk is the most extensively studied matrix for LAB-mediated folate bio-enrichment, with reported folate concentrations ranging from nanograms to micrograms per milliliter. Cucick et al. (2020) reported that milk fermented with *S. thermophilus* 34v and *L. plantarum* 16cv contained 321.1 ± 14.1 ng/mL of folate [9], corresponding to approximately 23% of the recommended daily intake for adults according to EFSA [69] and 20% (adults) to 40% (children) according to FAO/WHO [70]. Similarly, Czarnowska-Kujawska and Paszczyk (2021) observed a 59% increase in folate synthesis during seven days of storage when *L. delbrueckii* subsp. *bulgaricus*, *S. thermophilus*, and *B. bifidum* were used in combination [71].

Single-strain fermentations have also yielded significant folate levels, although results vary substantially. Divya and Nampoothiri (2014) reported a maximum folate concentration of 18.66 ± 0.36 µg/L after 8 h of fermentation with *L. lactis* CM28 [72], followed by a decrease attributed to folate utilization during microbial multiplication [17]. Jiao et al. (2020) demonstrated that milk fermented with *L. lactis* KLDS4.0325 at 37 °C for 24 h reached 30.59 µg/100 mL, significantly higher than non-fermented milk (3.01 µg/100 mL), in a murine folate-deficiency model; this fermented milk was as effective as synthetic folic acid in increasing 5-MTHF levels and reducing plasma homocysteine [73]. 

Fermentation temperature and co-cultivation strategies further influence folate accumulation. Milk fermented at 42 °C for 6 h with *L. bulgaricus* CRL871, *S. thermophilus* CRL803, and *S. macedonicus* CRL415 reached 187 ± 7 µg/L and prevented folate deficiency in rodents [63]. Additionally, among 14 tested *S. thermophilus* strains, folate production ranged from 27.12 ± 1.51 ng/mL to 76.61 ± 3.29 ng/mL after 48 h at 42 °C [11]. Wu et al. (2017) reported even higher values in milk fermented at 43 °C for 6 h, with *L. plantarum* ATCC 14,917 producing 63.23 µg/mL, followed by *L. casei* ATCC 15,008 (45.41 µg/mL) and *L. acidophilus* ATCC 4356 (42.78 µg/mL), differences attributed to the presence or absence of genes involved in the de novo folate biosynthesis pathway [74].

In contrast, some LAB strains act primarily as folate consumers. Mahara et al. (2021) observed reductions of 61.01%, 49.18%, and 27.35% in folate content when reconstituted skim milk was fermented with *L. fermentum* JK13, *L. plantarum* 4C261, and *L. rhamnosus* R23, respectively, resulting in final concentrations of 16.02, 20.88, and 29.85 ng/mL. These findings reinforce the strain-dependent nature of folate metabolism [17]. In line with this variability, Tidona et al. (2018) demonstrated that even among folate-producing LAB, production efficiency differs markedly. *Lactococcus hircilactis* LMG 28,352 synthesized 15.96 ± 0.86 µg/L of folate in unsupplemented skim milk, increasing to 29.97 ± 0.66 µg/L upon supplementation with 1.5 mg/mL pABA [75]. In contrast, *Lactococcus laudensis* LMG 28,353 did not accumulate detectable folate under either condition (0.59 ± 1.68 µg/L), indicating that even in the presence of a key biosynthetic precursor, this strain was unable to produce folate. Together, these results highlight that LAB may function as folate producers, non-producers, or net consumers, further emphasizing the importance of strain selection in folate bio-enrichment strategies.

As well as bovine milk, positive results were also obtained using UHT goat milk as substrate. Seven LAB isolates were used, of which five were *S. thermophilus* (FP 34, 170, 268, 341, and 361) and two *L. lactis* subsp. *lactis* (FP 343 and 368), and these were fermented at 37 °C for 24 h. All of them multiplied, reduced pH, and increased the initial folate content (40 ng/mL) to 92–313 ng/mL. *S. thermophilus* FP 268 achieved a nearly five-fold increase within 6 h, while the remaining isolates showed greater production after 8–24 h [76].

Yogurt production has also proven to be a favorable process for folate bio-enrichment. In one study, yogurt fermented with conventional LAB starters accumulated folate to concentrations ranging from 21 to 42 µg/L, confirming that the fermentation of dairy matrices can inherently support folate synthesis by suitable strains [77]. Building upon these findings, alternative formulations incorporating folate-producing strains or synbiotic strategies yielded even higher concentrations. When inulin was added, folate levels increased from 34.20 ± 1.10 µg/L to 57.80 ± 2.40 µg/L, while the inclusion of *Propionibacterium freudenreichii* resulted in final concentrations of 65.30 ± 3.10 µg/L, significantly exceeding those of conventional yogurt [78].

Cheese matrices allow folate accumulation during maturation. In goat milk cheese, *L. casei* VC199 increased folate concentration from 86.68 ± 4.74 µg/100 g after 30 days to 104.52 ± 2.24 µg/100 g after 60 days, while *L. paracasei* subsp. *paracasei* SE160 reached 122.58 ± 6.03 µg/100 g after 60 days [23]. In cow milk symbiotic cheese containing *L. salivarius*, *L. lactis*, and inulin, folate levels were 36.36 µg/g at day 0 and 3.97 µg/g after 28 days, whereas control cheese showed a rapid decline, with no detectable folate after day 14 [79].

In recent years, plant-based substrates have gained attention as vehicles for folate bio-enrichment. Fermented soy products containing passion fruit and fructooligosaccharides showed increased folate levels when inoculated with *S. thermophilus* ST-M6 and TH-4 [80]. Traditional cereal-based foods have also been explored: millet porridge (ben-saalga) fermented with *L. plantarum* 6.2 and *L. fermentum* 8.2 reached 7.1 and 7.3 µg/100 g, respectively, compared to 4.2 µg/100 g in traditionally prepared porridge although alternative processing methods did not always improve folate retention [81, 82].

Other cereal products also showed promising results. Quinoa noodles fermented with *L. plantarum* CRL 2107 and CRL 1964 reached folate levels of 1.6 ± 0.2 µg/g [83]. The role of *L. plantarum* in plant-based substrates for folate production was further demonstrated during the fermentation of a traditional sorghum-based food, *motoho*. In this preparation, five strains of *L. plantarum* (S7, S17, S27, S43, and S49) were inoculated into cooked and cooled porridge and fermented for 24 h at 30 °C. Strain S7 produced the highest folate content (20.0 ± 4.0 µg/100 mL), followed by S49 (19.0 ± 4.0 µg/100 mL), S27 (14.0 ± 4.0 µg/100 mL), and S43 (13.0 ± 3.0 µg/100 mL), whereas folate levels in S17 were below detection limits (< 2.5 µg/100 mL), using an HPLC method [84]. In addition, *L. plantarum* P2R3FA combined with *S. cerevisiae* increased folate content to 45.3 ± 1.7 µg/100 g in teff-based *injera* [85], while *L. rhamnosus* IMF4 enhanced folate concentrations to 42 ng/mL in ragi porridge [86].

Fruit, vegetable, and tuber substrates exhibited variable outcomes. Orange juice fermented with *S. thermophilus* NCIM 2904 reached 45.3 µg/100 mL, whereas tomato juice reached 33.56 µg/100 mL after 6 h of fermentation [87]. In contrast, apple juice fermented with *L. rhamnosus* LGG or *L. plantarum* L299v showed low folate levels (1.26 and 1.03 µg/100 mL) [88]. High folate concentrations were reported in fermented vegetable mixtures (up to 58.82 ± 1.98 µg/100 g), pumpkin leaves (269.9 ± 8.3 µg/100 g), tuber-based purees of *Solanum tuberosum* ssp. *andigena*, *Oxalis tuberosa* and *Ullucus tuberosus*, where *Lactobacillus sakei* strains produced up to 1484 ng/g and *tigernut* kefir where folate responses were variety-dependent: in the brown variety, folate decreased from 9.557 ± 0.17 to 9.125 ± 0.22 µg/100 mL, suggesting microbial consumption, whereas the yellow variety showed slight production (6.080 ± 0.11 µg/100 mL) without significant impact of fermentation on B-complex vitamins (*p* > 0.05) [89–92].

As summarized in Table 3, studies involving animal-based matrices (*n* = 15) generally report higher and more consistent folate accumulation than plant-based products (*n* = 14). Nevertheless, plant-based matrices offer relevant nutritional and technological advantages, particularly for populations with dietary restrictions. Overall, the literature confirms that LAB-mediated folate bio-enrichment is feasible across diverse food systems, provided that strain selection, substrate composition, and fermentation conditions are carefully optimized.

**Table 3:** Animal-based and plant-based products and respective lactic acid bacteria (LAB) strains used for folate production and determination method

Products	LAB	Folate content	Methodology	Reference
Animal-based				
Fermented bovine milk	*Streptococcus thermophilus* 34v and *Lactobacillus plantarum* 16cv	321.1 ± 14.1 ng/mL	MA	Cucick et al. (2020) [9]
Fermented milk	*Lactobacillus delbrueckii* subsp. *bulgaricus*,* Streptococcus thermophilus* and *Bifidobacterium bifidum*	60.3 ± 1.4 µg/kg	HPLC	Czarnowska-Kujawska et al. (2021) [71]
Fermented skim milk	*Lactococcus lactis* CM28	18.66 ± 0.36 µg/L	MA	Divya & Nampoothiri, (2014) [72]
Fermented milk	*Lactococcus lactis* KLDS4.0325	30.59 µg/100 mL	MA	Jiao et al. *(*2020) [73]
Fermented milk	*Lactobacillus bulgaricus* CRL 871, *Streptococcus thermophilus* CRL803 and *Streptococcus macedonicus* CRL415	187 ± 7 µg/L	MA	Laiño et al. (2015) [63]
Fermented milk	*Streptococcus thermophilus*	27.12 ± 1.51 ng/mL to 76.61 ± 3.29 ng/mL	MA	Meucci et al. (2018) [11]
Fermented milk	*Lactobacillus plantarum* (ATCC 14917), *Lactobacillus acidophilus* (ATCC 4356), and *Lactobacillus casei* (ATCC 15008)	63.23 µg/mL, 42.78 µg/mL and 45.41 µg/mL	HPLC	Wu et al. (2017) [74]
Fermented milk	*Lactobacillus fermentum* JK13, *Lactobacillus plantarum* 4C261 and *Lactobacillus rhamnosus* R23	16.02 ng/mL, 20.88 ng/mL, and 29.85 ng/mL	MA	Mahara, Nuraida & Lioe, (2021) [17]
Fermented milk	*Lactococcus hircilactis* and *Lactococcus laudensis*	15.96 ± 0.86 µg/L and 0.59 ± 1.68 µg/L	MA	Tidona et al. (2018) [75]
Fermented goat milk	*Streptococcus thermophilus* (FP 34, 170, 268, 341, and 361) and *Lactococcus lactis* subsp. *lactis* (FP 343 and 368)	220 ± 9 to 92 ± 14 µg/L and 115 ± 4 to 313 ± 81 µg/L	MA	Da Silva et al. (2016) [76]
Yogurt and fermented milk	*Lactobacillus bulgaricus* CRL 871, *Streptococcus thermophilus* CRL803 and *Streptococcus macedonicus* CRL415	Approx. 178 µg/L	MA	Laiño et al. (2013) [77]
Yogurt	*Propionibacterium freudenreichii* ssp. *Shermanii*	39.2 ± 0.7 µg/100 mL	HPLC	Zahed et al. (2022) [78]
Cheese from goat’s milk	*Lactobacillus casei* VC199 and *Lactobacillus paracase*i subsp. *paracasei* SE160	86.68 ± 4.74 µg/100 g and 122.58 ± 6.03 µg/100 g	MA	Albano et al. (2020) [23]
Cheese (Symbiotic cow’s milk cheese)	*Lactobacillus salivarius* and *Lactococcus lactis*	36.36 µg/g	HPLC	Arasteh et al. (2021) [79]
Plant-based				
Fermented soy product with passion fruit by-product and fructooligosaccharides	*Streptococcus thermophilus* TH-4 and *Lactobacillus rhamnosus* LGG	227 ± 0 ng/mL	MA	Albuquerque et al. (2019) [80]
Cereal-based fermented porridge *ben-saalga*	*Lactobacillus fermentum* 8.2 and *Lactobacillus plantarum* 6.2	7.3 µg/100 g and 7.1 µg/100 g	MA	Bationo et al. (2019) [81]
* Ben-saalga*	Autochthonous microorganisms	2.0 ± 1.1 µg/100 g	MA	Saubade et al. (2018) [82]
Fermented quinoa sourdough pasta	*Lactobacillus plantarum* CRL 2107 and *Lactobacillus plantarum* CRL 1964	1.6 ± 0.2 µg/g	MA	Carrizo et al. (2020) [83]
Fermented orange and tomato juice	*Streptococcus thermophilus* NCIM 2904	45.3 µg/100 mL and 33.56 µg/100 mL	MA	Deep, Ojha & Kundu, (2017) [87]
Apple, grape and orange juice	*Lactobacillus plantarum* 299v and *Lactobacillus rhamnosus* LGG	1.26 µg/100 mL and 1.03 µg/100 mL	HPLC	Espírito-Santo et al. *(*2015) [88]
African fermented sorghum gruel (*motoho*)	*Lactobacillus plantarum* (S7, S17, S27, S43, S49)	20.0 ± 4.0; ND; 14.0 ± 3.0; 13.0 ± 3.0 and 19.0 ± 4.0 µg/100mL	HPLC	Fayemi et al. (2023) [84]
* Teff injera* (Ethiopian fermented cereal food)	*Lactobacillus plantarum* P2R3FA and *Saccharomyces cerevisiae*	14.3 to 53.5 µg/100 g	MA	Tamene, Baye & Humblot, (2022) [85]
Ragi gruel	*Lactobacillus rhamnosus* IFM4	35 to 42 ng/ml	MA	Panda et al. (2018) [86]
Cauliflower and white beans	*Lactobacillus plantarum* (strain 299v, strain Lp900, strain 299, strain Heal19)	48.74 ± 3.98 to 58.82 ± 1.98 µg/100 g	MA	Thompson et al. (2020) [89]
* Curcubita* sp. leaves	Autochthonous microorganisms	211.1 ± 8.4 and 269.9 ± 8.3 µg/100 g	LC-MS/MS method	Misci et al. (2021) [90]
Tigernut (*Cyperus esculentos* L.)	*Water kefir grains*	6.080 ± 0.11 and 9.125 ± 0.22 µg/100 mL	MA	Satir, (2022) [92]
Potato *S. tuberosum* spp. a*ndigena*, oca *Oxalis tuberosa* and papalisa *Ullucus tuberosus*	*Lactobacillus sakei* CRL 2209 and CRL 2210	760 and 1484 ng/g	MA	Mosso et al. (2018) [12]
Cereal-based fermented food	*Lactobacillus*, *Weissella*, *Leuconostoc*, *Lactococcus* and *Pediococcus*	0 to 3.3 µg/100 g	MA	Saubade et al. (2017) [15]

*MA* Microbiological Assay, *HPLC *High Performance Liquid Chromatograph, *LC-MS/MS* Liquid Chromatography coupled to Mass Spectrometry, *ND* Not detecte

### Conclusion

An integrated analysis of the data, along with a meta-analysis, shows that research on the ability of LAB to synthesize folate represents an emerging scientific field, still in the consolidation phase, but with great technological potential. The recent concentration of publications (since 2020) reflects the growing interest of the scientific community, as well as the food industry, in sustainable biological strategies for the bio-enrichment of foods. The available results demonstrated that the application of folate-producing LAB capable of synthesizing folate in food matrices of both plant and animal origin is viable and enables the production of fermented foods with greater added value, aligned with contemporary demands for healthy, functional, and innovative products.

### Future Prospects

Based on the data collection and the meta-analysis conducted, it was confirmed that research investigating the capacity for folate synthesis by lactic acid bacteria is relatively recent; 2020 was the year with the most publications, indicating that this is a field with significant potential for further exploration. Generally, research focuses on the application of lactic acid bacteria with folate synthesis capacity in both animal and plant food matrices, with promising bio-enrichment results. These successful applications are commercially attractive, considering that the demand in the food market for healthy foods, including fermented foods that are not traditional, is increasing. The production of fermented dairy and non-dairy products bio-enriched with folate through the fermentation process by lactic acid bacteria can be achieved in the medium term by industries.

Furthermore, the production of bio-enriched foods with folate can help reduce risks and manage health challenges associated with low consumption, both nationally and globally. Thus, scientific and technological capacity for sustainable use, production, and consumption is strengthened, allowing the use of natural resources with resilience and equity, promoting the circular economy, and reducing environmental impacts.

It is suggested that future studies prioritize the study of individual strains whose folate synthesis is already established, further elucidating the regulation of key genes in the folate biosynthetic pathway through integrative omics approaches (combining genomics, transcriptomics, proteomics, and metabolomics). This would contribute to understanding existing bottlenecks regarding regulatory mechanisms, as well as stress response mechanisms and metabolic fluxes that enable and drive high folate yields under conditions relevant to food production.

Another critical viewpoint with a technological bias that could be further explored would be the optimization of fermentation parameters, since the systematic evaluation of the composition of the substrates/food matrices used, as well as the availability of precursors, pH, temperature, and also the control of the growth phase, can provide an increase in the ability to replicate endogenous folate production results without compromising microbial viability or food quality.

Regarding nutrition and public health, a significant gap was identified in the number of clinical and dietary intervention studies capable of evaluating the bioavailability and metabolic fate of folate produced by LAB in humans, particularly in vulnerable groups at risk of folate deficiency. This type of data would be fundamental to supporting evidence-based health claims and guiding regulatory frameworks.

## Data Availability

No original data were used in this study, as this is a review article. All data discussed are found in existing publications, duly referenced in this manuscript.
